# Epidemiology and Control of Meningococcal Disease in Canada: A Long, Complex, and Unfinished Story

**DOI:** 10.1155/2019/8901847

**Published:** 2019-11-25

**Authors:** Philippe De Wals

**Affiliations:** ^1^Department of Social and Preventive Medicine, Laval University, Quebec City, Canada; ^2^Centre de Recherche de l'Institut de Cardiologie et de Pneumologie de Québec, Quebec City, Canada; ^3^Centre de Recherche du Centre Hospitalier Universitaire de Sherbrooke, Sherbrooke, Canada; ^4^Institut National de Santé Publique du Québec, Quebec City, Canada

## Abstract

The epidemiology of meningococcal disease in Canada has been punctuated by outbreaks caused by serogroup A strains in the 1940s, virulent serogroup C clones from 1985 to 2001, a serogroup B clone in Quebec from 2003 to 2014, and more recently a W clone in British Columbia. Region- and province-wide immunization campaigns have been implemented to control these outbreaks using meningococcal C polysaccharide and conjugate vaccines, a quadrivalent ACWY conjugate vaccine, and a serogroup B protein-based vaccine. Meningococcal C conjugate vaccines have been included in routine immunization programs for children, and ACWY conjugate vaccines have been included in school-based programs for adolescents in most jurisdictions. In contrast, serogroup B protein-based vaccines were only recommended and used for high-risk individuals and to control outbreaks. Currently, the immunization schedules adopted in provinces and territories are not uniform. This is not explained by notable epidemiologic differences. Publicly funded immunization programs are the result of a complex decision-making process. Political factors including public opinion, media attention, interest groups' advocacy campaigns, decision-makers' priorities and budgetary constraints have played important roles in shaping meningococcal programs in Canada, and this should be recognized. As the recent occurrence of outbreaks caused by virulent W clones shows, continued investments in epidemiological surveillance at both the provincial and national levels are necessary, so there can be early warning and informed decisions can be made.

## 1. Introduction

Invasive meningococcal disease (IMD) in Canada is characterized by its unpredictability and severity. Five *Neisseria meningitidis* (*N.m.*) serogroups (A, B, C, W, and Y) cause most invasive diseases worldwide [1]. In North America, the majority of cases are sporadic, but outbreaks of variable magnitude may occur. It is one of the most feared diseases by the population and by clinicians and garners a large amount of media attention. In the last century, substantial efforts have been made to develop meningococcal vaccines [2, 3]. Currently, monovalent serogroup C conjugate (MenC-Con) and quadrivalent serogroup ACWY conjugate (MenACWY-Con) vaccines are included in publicly funded immunization programs for children and adolescents in Canada, whereas serogroup B protein vaccines (MenB-Prot) are only offered to high-risk individuals and to control outbreaks [4, 5]. The objective of this article is to tell the story of the epidemiology of invasive meningococcal disease (IMD) and immunization programs in Canada, starting in 1940. Prospects for future developments in immunization programs are discussed. Special attention is paid to the immunization strategies and the factors that influenced the decisions to launch targeted immunization campaigns and routine immunization programs for children and adolescents. The applicability of different elements of the “Multiple Stream Frameworks” proposed by John Kingdon to explain how governmental policies are decided is illustrated [6, 7].

## 2. Serogroup A Epidemic in the Early 1940s and Polysaccharide Vaccine Development

On September 10, 1940, King George VI approved Canada's declaration of war with Germany and soon after, the armed forces were mobilized and there was a call for voluntary service [8]. The influx of military recruits in barracks triggered a number of IMD outbreaks caused by serogroup A strains [9, 10]. The incidence peaked in 1941 with a rate of 12.7 per 100,000 population [11]. At that time, no vaccine was available and outbreaks triggered large chemoprophylactic interventions using sulfonamides, but resistance emerged [12]. In the 1960s, a major US military research program was launched and resulted in the development of the first meningococcal polysaccharide vaccines [13]. Bivalent (serogroups A and C), trivalent (serogroups A, C, and W), and quadrivalent (serogroups A, C, W, and Y) polysaccharide vaccines were authorized in Canada in the 1990s, but they were mainly used to protect military recruits and were recommended for travelers visiting high-risk areas outside Canada [14].

## 3. Serogroup C Outbreaks and Immunization Campaigns with Polysaccharide Vaccines in 1985–1992

After the end of the World War II, an endemic situation prevailed until 1985 with yearly incidence rates ranging between 0.4 and 2.0 per 100,000 population [11]. Serogroups A and C were most frequently identified until 1975, after which serogroup B predominated. In 1986, a steady increase in IMD incidence started, caused by the emergence of a virulent serogroup C clone of electrophoretic type ET-15 (classified later in the ST-11 clonal complex) [11]. The Atlantic provinces and Quebec were predominantly affected, as well as Ontario to a lesser extent. Serogroup C ET-15 IMD cases tended to occur in clusters with an unusual proportion of cases among adolescents and young adults. Results from a Quebec study showed that up to 80% of serogroup C cases had signs of septicemia, of which 14% resulted in fatality, and of the survivors, 15% had physical sequelae that mainly consisted of skin scars and amputations [15]. These outbreaks led to immunization campaigns using polysaccharide vaccines in Prince Edward Island, Ontario, and Quebec.

In Prince Edward Island (PEI), eight confirmed and suspected serogroup C IMD cases occurred between December 1990 and January 1992, whereas only two serogroup B cases had been reported from 1980 to 1989 [16]. This situation generated a lot of anxiety and media attention. In December 1991, public health authorities decided to offer the vaccine to approximately 1,000 people in a high school where one student died from the disease. Instead of calming the anxiety, this decision added fuel to the fire. After the occurrence of another IMD case in January 1992, public health services were flooded with demands for immunization. Following a consultation with Health Canada and provincial health authorities, the PEI Ministry of Health decided to offer the vaccine to all 54,000 residents aged 2 to 29. The mass immunization campaign was launched on January 15, 2002, with 91% of the target group receiving an ACWY polysaccharide vaccine. Eventually, the outbreak was controlled.

Another highly publicized cluster of serogroup C cases occurred in the Ottawa-Carleton region of Ontario and in the adjacent Gatineau region of Quebec during the winter of 1991–1992 [17–19]. There was heavy media coverage and local public health services were soon flooded with demands for intervention. In January 1992, a mass immunization campaign using an ACWY polysaccharide vaccine was launched in the Ottawa-Carleton region, and approximately 145,000 residents 6 months to 20 years of age were vaccinated for an estimated total cost of $2.6 million. No other significant clusters were reported in the other regions of Ontario at that time.

In Quebec, serogroup C sporadic cases and clusters occurred with increased frequency starting in 1986 and small-scale vaccinations were carried-out during the winter and spring of 1991-1992 [20]. About 300,000 doses of polysaccharide vaccines were administered in different settings, but these interventions were not successful in preventing the spread of the virulent clone. Thus, the decision was made to implement a province-wide immunization campaign targeting all residents between 2 months and 20 years of age. At the end of the campaign that took place during the winter of 1992-1993, about 1.6 million doses of vaccines had been administered, covering 84% of the target population. In the first year following the campaign, the incidence of serogroup C IMD showed a marked drop among vaccinees as well as the unvaccinated fraction of the target population, while remaining unchanged among people over 20 years of age. This suggested the existence of a horizontal component of herd immunity (protection in the same age stratum) but not a vertical one (protection of older people). A cohort analysis was undertaken to assess vaccine effectiveness (VE) [21]. Protection against serogroup C IMD was demonstrated in the first 2 years after vaccine administration (VE: 65%; 95% CI: 20%–84%), but not in the following 3 years (VE: 0%; 95% CI: –5%–65%). VE was strongly related to the age at the time of vaccination: 83% (95% CI: 39%–96%) for ages 15 through 20, 75% (95% CI: 17%–93%) for ages 10 through 14, and 41% (95% CI: 106%–79%) for ages 2 through 9. There was no evidence of protection in children younger than 2 years of age; all 8 MCD cases in this age group occurred in vaccinees. A *post hoc* economic evaluation of the campaign in Quebec was performed [22]. The average cost of purchasing vaccines was reasonable ($5.26 per dose), and the overall cost of the campaign to the health system was $26 million. Between 48 (assuming no herd effect) and 74 CMD cases (assuming some herd effect) were prevented in the following 5 years. Net societal costs of the campaign were between $18 million and $21 million (using a 3% discount rate) and between $49,000 and $87,000 per quality-adjusted life-year (QALY) gained.

## 4. Serogroup C Outbreaks in 1999–2001 and First Conjugate Vaccines

Another outbreak caused by a virulent serogroup C ET-15 (ST-11) *N.m.* clone started in the Edmonton area, Alberta in December 1999 and spread to adjacent regions in 2000 [23, 24]. The IMD incidence peaked in 2000 with a rate of 2.5 per population of 100,000, versus the rate of 1.0 per 100,000 seen in previous years. To control this outbreak, an immunization campaign using a quadrivalent polysaccharide vaccine was first implemented in the Capital Health region in February 2000 and was progressively extended to the whole province. At the end of the campaign in March 2002, 76% of approximately 750,000 residents 2 to 24 years of age were immunized. The overall effectiveness of the vaccine was estimated to be 84% [23]. In April 2002, Alberta was the first province to introduce a recently authorized MenC-Con for the routine immunization of infants (initially, 3 doses were offered, one each at 2, 4, and 6 months of age).

The outbreak that started in the Quebec City region in February 2001 was caused by a parent serogroup C ET-37 (ST-11) *N.m.* clone [25, 26]. First, localized interventions using plain polysaccharide vaccines that targeted secondary school students were conducted. However, the epidemic extended to younger age groups and other regions, which generated a high level of anxiety in the population and resulted in extensive media coverage. At the request of the Quebec Ministry of Heath, a first serogroup C oligosaccharide-CRM_197_ protein conjugate vaccine was authorized by Health Canada following a fast-track process, and a mass immunization campaign was launched, starting in the Quebec City area in May 2001. The campaign was extended to other regions during the autumn of 2001, targeting all residents in the province between the ages of 2 months and 20 years. At the end of the campaign, 82% of the target group of 1.9 million people was immunized, and the outbreak was successfully controlled. Incidence of meningococcal C disease markedly decreased in not only highly vaccinated but also in poorly vaccinated and nonvaccinated birth cohorts, suggesting a herd effect. Overall vaccine effectiveness was 87.4% (95% CI: 75.4%–94.2%) with lower protection in children vaccinated under 2 years of age and a higher magnitude of waning protection in this age group. At that time, the purchase cost of MenC-Con was about $50 per dose as only one manufacturer was able to provide the large number of doses requested [27]. No *post hoc* economic analysis was performed. In the fall of 2002, MenC-Con was introduced to the routine immunization schedule of children, with one dose offered at 12 months of age.

## 5. Routine Immunization Using Serogroup C Conjugate Vaccines

In October 2001, the National Advisory Committee on Immunization issued a first statement on the first authorized MenC-Con [28]. For the routine immunization of infants, a total of 3 doses were recommended, one each at 2, 4, and 6 months of age. One dose was recommended for people one year of age and older. Subsequent statements were issued when new vaccines were authorized [29, 30]. All Canadian provinces and territories progressively introduced MenC-Con for the routine immunization of infants using different schedules (3 + 0, 2 + 1, 1 + 1, or 0 + 1 primary + booster doses). Also, primary catch-up vaccinations were offered to different age groups and in routine school-based programs in most jurisdictions. Eventually, revaccinating adolescents who had been immunized during childhood was recommended in light of immunogenicity and effectiveness data that indicated waning protection when vaccinated at an early age [31]. In order to ensure herd effect, there was the need to vaccinate adolescents due to the fact that they are the main reservoir for transmitting *N.m.* in the population [32]. All of these decisions were undoubtedly influenced by the risk of further serogroup C outbreaks like the one observed in the Abbotsford area of British Columbia in 2001 [33]. Two model-based studies published in 2004 and 2006 demonstrated that a program consisting of one MenC-Con toddler dose and a booster dose in adolescence was an effective control strategy that would generate acceptable cost-effectiveness indices, even with a vaccine purchase price of $50 per dose [27, 34]. Marketing three MenC-Con products also brought down vaccine prices in tenders proposed to the public sector (Table 1). Finally, the introduction of MenC-Con was facilitated to some extent by a 2004 initiative by the Government of Canada to provide $400 million (in the form of a trust fund) to help start or expand provincial and territorial immunization programs that combat chicken pox and pneumococcal and meningococcal diseases [35].

The high uptake of MenC-Con vaccinations among both young children and adolescents certainly contributed to a marked decrease in the incidence of serogroup C IMD in Canada. According to the 2017 Childhood National Immunization Coverage Survey, 88% of Canadian children had received at least one dose of meningococcal vaccine by their second birthday [36]. This may be an underestimation of the true uptake. In Quebec, results of the methodologically more valid 2016 provincial immunization survey showed that 95% of children had received at least one dose of meningococcal vaccine by their second birthday [37]. For the adolescent MenC-Con dose offered in Grade 9, the 2018 estimate from the Quebec Provincial Immunization Registry is 87% (Kiely M, written communication). Before MenC-Con introduction in Canada, serogroup C disease was 0.07–0.25 per 100,000 per year depending on the province and was below 0.05 per 100,000 per year in 2012, with a majority of cases in unvaccinated adults [38]. This is a success story!

## 6. Adoption of Quadrivalent Conjugate Vaccines for Adolescents from 2006 to 2016

The first quadrivalent MenACWY-Con was authorized in Canada in 2006. Two other products came, respectively, in 2010 and in 2013 (Table 1). In a statement published in 2007 [39], the National Advisory Committee on Immunization concluded that vaccinating against serogroups A, Y, and W would cause only a slight decrease in the health burden of IMD, given that these serogroups were relatively uncommon. It also concluded that the use of MenACWY-Con should be considered only for individuals or circumstances where serogroups A, Y, or W frequently occur. A more permissive statement was published in 2009 specifying that the adolescent dose could be provided using monovalent meningococcal C conjugate vaccine or quadrivalent conjugate meningococcal vaccination for A, C, Y, and W depending on local considerations (i.e., serogroup-specific incidence, age distribution of cases, and vaccine price) [40]. This change was not justified by a substantial epidemiologic change in Canada or new effectiveness data but was in line with the recent adoption of MenACWY-Con for adolescents in Prince Edward Island and New Brunswick. An analysis of IMD surveillance data in Canada from 2006 to 2011 showed no serogroup A cases and a low incidence of serogroup Y and W IMD at 0.10 and 0.03 per 100,000 people per year, respectively, with no evidence of upward trends [41]. The severity of Y and W IMD cases was lower than that of serogroup C cases at that time [42]. A model-based study published in 2015 suggested that a very effective control strategy would be to administer one MenACWY-Con dose at 12 months of age followed by an adolescent booster dose, but the cost was not considered [43]. An economic evaluation published in 2017 showed that in Canada, adopting MenACWY-Con instead of MenC-Con for the immunization of adolescents would result in modest health benefits at a high cost, reaching acceptable incremental cost-effectiveness ratios only in scenarios with much higher ACWY disease incidence or lower MenACWY costs (the differential vaccine cost was $12/dose in the base model) [44]. A survey conducted among registered nurses in Quebec in 2008 and one conducted with Canadian pediatricians and family physicians in 2009 showed that the introduction of MenACWY-Con was not listed as a priority when compared with other new vaccines and programs [45, 46].

Nevertheless, MenACWY-Con was introduced for the immunization of adolescents in Prince Edward Island and New Brunswick in 2008. In these two provinces combined, the reported number of cases of either serogroup Y or W during the period 1995–2006 was one per year [40]. MenACWY-Con was progressively introduced to school-based adolescent programs in other jurisdictions, although not in Quebec, due to cost-effectiveness considerations and local epidemiology [44]. Several reasons may explain these decisions: temporary increases in W or Y rates in some jurisdictions, the important role of NACI recommendations (which are less influential in Quebec), the absence of cost-effectiveness analyses in most provinces, the media attention towards a few fatal cases, the advocacy role of parents, and the influence of pharmaceutical companies.

## 7. Serogroup B Vaccines and Outbreaks in Quebec, 2003–2016

It took many years to develop vaccines against serogroup B meningococcus [47]. The first protein-based meningococcal vaccine (4CMenB) was authorized in 2013 and a second one (MenB-FHbp) in 2018 (Table 1). These vaccines were licensed on the basis of immunogenicity and safety data without any information on their efficacy or effectiveness [48]. Both the NACI recommendations for health professionals and the Pan Canadian Public Health Network recommendations for provincial and territorial public health authorities were restrictive, limiting the use of 4CMenB to high-risk individuals and to control outbreaks [49, 50]. Similar recommendations were issued by provincial expert committees [51, 52]. Although many clinicians and health professional organizations advocated more liberal use of these protein-based vaccines [53–55], public health specialists expressed many concerns: the low burden of disease, the uncertain duration of protection and effect on carriage, the reactogenicity of 4CMenB, the budget impact and unfavorable cost-effectiveness indices, and the operational difficulties in administering additional vaccines in an already crowded infant immunization schedule [56, 57].

In 2003, a virulent serogroup B ST-269 *N.m.* clone emerged in Quebec and spread throughout the province [58]. Strains from this clone had surface proteins close to the antigens included in 4CMenB. The Saguenay-Lac-Saint-Jean (SLSJ) region was particularly affected with an average annual incidence rate of 3.4 per 100,000 person-years from 2006 to 2013. In May 2014, an immunization campaign was launched in this region, using the newly authorized 4CMenB [59]. By the end of the campaign, 83% of the 59,000 SLSJ residents between 2 months and 20 years of age had been vaccinated. However, vaccine uptake was lower (47%) in the 17–20-year-old age group [60]. Active surveillance for adverse events confirmed the safety of the vaccine, although local reactions and fever were frequently observed [61]. Five serogroup B IMD cases occurred in the SLSJ region from July 2014 to June 2018, including in one vaccinated child. Direct vaccine protection was estimated to be 79% (95% CI: 231%–99%), and there was no evidence of herd effect among unvaccinated adults [62]. Another cluster of serogroup B cases occurred in a small area to the south of Quebec City from 2015–2016, which triggered another intervention targeting children under 5 years of age [63]. The ST-269 outbreak has now subsided in the province of Quebec, and the use of 4CMenB has been discontinued in the two most affected regions. Incidentally, the Quebec Ministry of Health did not release the cost of these campaigns, citing commercial and trade secrets. This decision was challenged by researchers in bioethics who argued that there is a moral and legal obligation to disclose major governmental expenses [64]. The Quebec Information Access Commission ruled in favor of this request, and the total vaccine purchase cost of $10.2 million for 130,000 doses was finally disclosed [65]. No *post hoc* cost-effectiveness evaluation of the campaign was performed.

## 8. Emergence of Serogroup W ST-11 Clone and Outbreak in British Columbia, 2014–2018

In recent years, virulent *N.m.* W ST-11 clones caused epidemics in South America and Europe that were characterized by a widespread distribution in age, atypical clinical presentations (e.g., gastrointestinal signs), and a high case-fatality rate [66]. One of these clones emerged in Canada in 2014 and spread throughout the country [67]. It caused a small outbreak of ten cases in the Okanagan region of British Columbia in 2017 [68]. In British Columbia, the school-based program for adolescents in Grade 9 including MenACWY-Con was introduced in the fall of 2016, meaning that most of the population was not immunized against serogroup W clones at the start of this outbreak. To control the outbreak, an immunization campaign was immediately launched, targeting 14–19-year-old students in the Okanagan region. At the end of the campaign, more than 14,000 individuals had received MenACWY-Con, and no further cases were reported in the region in 2018 (Monika Naus, written communication). The clone is now present in other regions of Canada including Quebec, and there is a potential for other outbreaks [69].

## 9. Current Meningococcal Vaccine Programs and Perspectives

Currently, two MenC-Con, three MenACWY-Con, and two MenB-Prot are authorized and distributed in Canada (Table 1). The programs implemented in Canadian provinces and territories (as of August 31, 2019) are described in Table 2. MenC-Con is used for infants and toddlers in all jurisdictions. MenACWY-Con is used for older children and adolescents in school-based program targeting students in Grade 4 to 12, according to provinces/territories. Quebec is an exception, and MenC-Con is still used in Grade 9. The incidence of serogroups W and Y is still low in this province, and following on an economic evaluation, the adoption of a quadrivalent was not recommended [70]. Any substantial increase in the incidence of serogroup W disease or an outbreak as observed in British Columbia could rapidly change this. In Canada, the number of cohorts of adolescents who have been immunized with MenACWY-Con ranged from 11 in Prince Edward Island and New Brunswick (starting in 2008) to zero in Quebec. This may be insufficient in preventing further outbreaks caused by a virulent serogroup W ST-11 clone.

A routine immunization program is good for preventing outbreaks in the long run but not for the rapid control of an emerging outbreak, and a mass campaign is needed for this. Ideally, interventions in the ascending phase of an outbreak are recommended to maximize the effectiveness of the campaign in terms of direct protection and herd effect. Also, selecting the target age group is especially difficult when there are cases in young infants, adolescents, and the elderly and lack of information on carriage. The dilemma experienced in many countries facing the emergence of the virulent ST-11 W *N.m.* clone is choosing between a strategy aimed at interrupting transmission by targeting adolescents and young adults and a strategy aimed at providing direct protection to all affected age groups, which is a safer but more expensive option [66].

Also, there is variation in the recommended immunization schedule for young children in different jurisdictions (Table 2). Although more doses in early childhood provide higher antibody levels [71], there is no much evidence of a difference in the long-term impact of different schedules [48]. Accordingly, a one toddler dose + one adolescent dose of meningococcal conjugate vaccine as now recommended in the UK is probably the most cost-effective strategy [72].

Because there is always the risk of outbreaks caused by virulent A, C, W, and Y *Neisseria meningitidis* (*N.m.*) clones, a prime toddler + adolescent boost approach using a quadrivalent meningococcal conjugate vaccine would be a prudent strategy to recommend when the price difference between monovalent and quadrivalent products is low. Several vaccines may be used for this, but there are differences in their immunologic response in young children and in their respective authorization. MenACWY-Con-DT is authorized for use in persons 9 months to 55 years of age, with two doses at least three months apart recommended for those 9 to 23 months of age [73]. MenACWY-Con-CRM197 is authorized for use in persons 2 months to 55 years of age, with two doses at least two months apart recommended for those 7 to 23 months of age [74]. MenACWY-Con-TT is authorized for use in persons 6 weeks to 55 years of age with a single dose recommended for those aged 12 months or more [75]. Clinical trials with MenACWY-Con-TT administered to 12–23-month-old infants showed that a single dose was highly immunogenic and that bactericidal antibody levels against serogroup C *N.m.* were noninferior to those measured after a single dose of MenC-Con-CRM197 [76]. Antibody levels decrease with time, and epidemiologic studies have shown that the protection against serogroup C IMD in toddlers who received one MenC-Con dose rapidly waned [77]. Using MenACWY-Con instead of MenC-Con in infants or toddlers would provide short-term protection against the three additional serogroups, but more importantly, it would prime the immune system and enable a more intense memory response following an adolescent booster dose as observed with MenC-Con [78]. Results from a recent immunogenicity study showed that primary vaccination of infants/toddlers with MenACWY-Con resulted in moderate antibody persistence for up to 4 years after the last priming dose, but a booster dose at 60 months of age induced a robust immune response against all serogroups [79].

The likelihood of adopting MenB-Prot into routine programs for children and adolescents in Canada is more doubtful. The 4CMenB vaccine has been shown to provide direct protection against serogroup B IMD both in the UK (3-dose infant-toddler program) and in Quebec (mass immunization campaign targeting individuals 2 months to 20 years of age) [62, 80]. In South Australia, the current immunization schedule includes three 4CMenB doses offered, respectively, at 2, 4, and 12 months of age and 2 doses for adolescents [81]. This was justified by the high B IMD rate in South Australia compared to other Australian states. However, inducing a herd effect through a program aimed at adolescents is uncertain. In two trials in the UK and South Australia, respectively, 4CMenB showed little effect on the prevalence and acquisition of asymptomatic carriage of *N.m.* [82–84]. In Quebec, no herd effect was observed following a mass immunization campaign in which more than 90% of individuals less than 17 years of age and 47% of those aged 17 to 19 years were immunized [62]. Adding at least three injections with a reactogenic vaccine, especially when administered with other vaccines, in an already crowded vaccination schedule would also be problematic. Finally, other deterrents include the substantial budgetary impact and the fact that infant programs are not cost-effective due to the low incidence of serogroup B IMD in most Canadian jurisdictions [85].

## 10. Decision Making and the Applicability of the Kingdon's Model

The investigation of decision processes regarding immunization programs in Canada is particularly challenging. The rationale of recommendations issued by the National Advisory Committee on Immunization (NACI) is presented in statements published on the Public Health Agency of Canada's website. Decisions regarding the implementation of publicly funded program are made at provincial and territorial levels, however [86]. The only province that produces a full report on the rationale for any important decision is Quebec, and they are published on the “*Institut national de santé publique du Québec*” website [87]. In other provinces, decisions are usually announced in short documents and/or press releases briefly mentioning the reasons, and two examples are given in Table 3 [88, 89]. Evidently, all new programs/changes are incorporated into provincial immunization manuals, and extensive references to NACI statements and the Canadian Immunization Guide are provided in these documents.

Implementing a publicly funded immunization program is the result of a complex decision-making process. John Kingdon's “Multiple Streams Framework” has been extensively used in order to analyze how and why governmental policies were adopted [6, 7]. According to this analytical framework, ideas that will ultimately end up in a proposal for a new immunization program develop gradually along three main streams: (i) the problem stream, which focuses on a particular vaccine-preventable disease and its perception by stakeholders; (ii) the policy stream, which is centered on experts' views on the optimal use of available vaccines; and (iii) the politics stream, which consists of sociopolitical factors, including budgetary constraints. Ideas are progressively shaped by policy entrepreneurs into a proposal with concrete implementation strategies. The three streams then converge within a policy window, during which adoption is especially likely to occur. To survive, the proposed program should be operationally feasible, consistent with mainstream social values, and financially affordable. The timing of the policy window is usually unpredictable and of short duration.

As described in this manuscript, the adoption of the MenC-Con was mostly due to the occurrence of outbreaks and the threat of future ones. As stated by Kingdon [6], an important distinction must be made between the objective assessment of the magnitude of a problem and how it is perceived by experts, clinicians, public health authorities, the population, and the media. Slovic [90] proposed a theory on the perception of risk which has been successfully applied to environmental hazards. In this theory, the perception of risk associated with a particular technological hazard and the willingness to invest resources in prevention measures are influenced by two main characteristics: the absence of knowledge regarding the hazard (not observable, unknown to those exposed, delayed effect, new risk, and risk unknown to science) and the dreadful character of the hazard (uncontrollable, dreaded, catastrophic and fatal consequences, not equitable, risk to future generations, not easily reduced, and involuntary). These characteristics apply to invasive meningococcal disease occurring in an unpredictable way in mostly healthy individuals and with severe consequences [91]. For these reasons, meningococcal vaccines are among those with the highest visibility and level of support in the population and among health professionals, although the absolute number of cases identified during and outside outbreaks remains low [46, 92]. Ultimately, cost-effectiveness studies that were performed for MenC-Con [27] seemed to have little importance in final decisions.

Another example of the role of nontechnical factors is the adoption of MenACWY-Con instead of MenC-Con for programs targeting adolescents. In BC, the decision was justified by the occurrence of serogroup W and Y cases in older teens and young adults as described in Table 2 [88]. This was however not seen in all provinces and territories. The availability of quadrivalent vaccines and the influence of vaccine manufacturers may have played a role. Pharmaceutical companies are promoting their products, and this is legitimate and lawful. A large array of tools is available for this, including the support of educational, surveillance and research activities, the mobilization of professional and family organizations, the lobbying of public health and political authorities, and the design of contracts including both essential and nonessential vaccines. In a thorough analysis of the role of industry in shaping state human papilloma virus immunization policymaking in the US, it was found that legislators relied heavily on pharmaceutical companies for information, especially in states with lean staff resources and expertise [93]. For protein-based meningococcal vaccines, the decision-making process was unusually conducted in a rational way and relied on carefully conducted evaluations, but this is exceptional in the history of meningococcal vaccines in Canada [49–52].

## 11. Conclusion

Canada is a very large country with six time zones and a distance of 7,200 km between St-Johns in Newfoundland and Victoria in British Columbia, with diverse climatic conditions ranging from mild maritime influences to the harsh arctic environment and a diversity of lifestyles influenced by socioeconomic and cultural factors. It is thus not surprising that the epidemiology of meningococcal disease varies between regions, as described in this manuscript. In the Canadian health system, immunization programs are primarily decided, funded, and implemented by provincial and territorial governments according to their own priorities. The roles of the federal government are to authorize vaccines and control their quality, make recommendations for vaccine use, and propose common goals and objectives for immunization programs. The common objectives of immunization programs targeting meningococcal disease that are currently proposed by the Public Health Agency of Canada are defined in terms of vaccine uptake and the reduction of disease burden (i) to achieve 95% vaccination coverage by two years of age for one dose of meningococcal C vaccine and 90% coverage by 17 years of age for one dose of meningococcal vaccine and (ii) to maintain a rate of less than five cases of serogroup C IMD per year in children less than 18 years of age [94]. All of aforementioned factors explain why programs are not uniform across the country.

As discussed in this manuscript, it should be recognized that political factors including public opinion, media attention, interest groups' advocacy campaigns, decision-makers' priorities, and budgetary constraints played important roles in shaping meningococcal programs. The idea that technical criteria such as an incremental cost-effectiveness threshold are the only valid arguments that can be used to justify immunization programs does not hold under scrutiny.

## Figures and Tables

**Table 1:** Meningococcal conjugate and protein vaccines authorized for marketing and sale in Canada.

Brand name	Current distributor	Composition: polysaccharide and carrier protein	Acronym used in manuscript	Authorization date
Menjugate™	GlaxoSmithKline	C with CRM_197_	MenC-Con (CRM)	September 2001
Neisvac-C™	Pfizer	C with TT	MenC-Con (TT)	January 2002
Meningitec™	Nuron Biotech	C with CRM_197_	MenC-Con (CRM)	February 2004^*∗*^
Menactra™	Sanofi Pasteur	ACWY with DT	MenACWY-Con (DT)	October 2006
Menveo™	GlaxoSmithKline	ACWY with CRM_197_	MenACWY-Con (CRM)	July 2010
Nimenrix™	Pfizer	ACWY with TT	MenACWY-Con (TT)	August 2013
Bexsero™	GlaxoSmithKline	NHBA, NadA, fHbp, and OMV (PorA P1.4)	Men-Prot or 4CMenB	December 2013
Trumenba™	Pfizer	Bivalent FHbp	Men-Prot or MenB-FHbp	July 2018

^*∗*^Withdrawn from marketing and authorization suspended in April 2014 following a problem with the production process.

**Table 2:** Routine immunization programs for children and adolescents (school-based programs) in Canadian provinces and territories (as of August 31, 2019).

Jurisdiction	Infants/young children		Older children/adolescents	
	Age	Vaccine	Grade^*∗*^	Vaccine
*Provinces*
British Columbia	2, 12 months	MenC-Con	9	MenACWY-Con
Alberta	4, 12 months	MenC-Con	9	MenACWY-Con
Saskatchewan	12 months	MenC-Con	6	MenACWY-Con
Manitoba	12 months	MenC-Con	9	MenACWY-Con
Ontario	12 months	MenC-Con	7	MenACWY-Con
Quebec	12 months	MenC-Con	9	MenC-Con
New Brunswick	12 months	MenC-Con	9	MenACWY-Con
Nova Scotia	12 months	MenC-Con	7	MenACWY-Con
Prince Edward Island	12 months	MenC-Con	9	MenACWY-Con
Newfoundland &Labrador	12 months	MenC-Con	4	MenACWY-Con

*Territories*				
Yukon	2, 12 months	MenC-Con	9	MenACWY-Con
Northwest Territories	2, 12 months	MenC-Con	12^*∗∗*^	MenACWY-Con
Nunavut	12 months	MenC-Con	9	MenACWY-Con

^*∗*^School-based program; ^*∗∗*^vaccine offered only to those grade 12 students who will be attending postsecondary education outside of the NWT.

**Table 3:** Examples of information issued by provincial health authorities in Canada regarding changes in immunization programs.

Purpose	Information	Reference
Introduction of MenACWY-Con for adolescents in British Columbia in 2016	*Q: why has the school-based pogram changed from Msn-C-C-C program to a MenC-ACYW-135 program?A: BC is replacing the grade 6 MenC-C program with a grade 9 MenC-ACYW-135 program to provide broader protection against meningococcal disease, including that caused by serogroup Y. This quadrivalent vaccine will continue to offer protection against serogroup C, which caused outbreaks in school-age children and young adults in several provinces including BC prior to introduction of the MenC-C vaccine. It will also offer protection against serogroup Y, which has resulted in fatal cases in BC in older teens and young adults. From 2001–2015, 23 cases including 4 deaths from serogroup Y meningococcal disease were reported in BC in those aged 15–24 years. While serogroup W-135 has caused only 3 cases and no deaths in this age group in the same period of time and serogroup A does not occur in North America, these two strains do occur more frequently in other parts of the world and the vaccine will offer this additional protection.*	BC Centre for Disease Control, 2016.

Introduction of MenACWY-Con for adolescents in Manitoba in May 2019	*Q*: *when did Manitoba's immunization program start?R*: *Meningococcal Immunization Program: Started in 2004 and offered in grade 4. The vaccine was not offered in 2017 and 2018 while it transitioned from grade 4 to grade 6. Starting September 2019, meningococcal conjugate quadrivalent (MenC-ACYW-135) vaccine will be offered in grade 6 for those born during or after 2008. No child will have missed the opportunity to be immunized against meningococcal disease.Q*: *how are decisions made to fund vaccines in Manitoba?R*: *Manitoba Health, Seniors and Active Living (MHSAL) gathers evidence-based information from a variety of sources, including current research and data (e.g., epidemiology, vaccine safety and effectiveness data, cost-effectiveness data, etc.), programs in other provinces and territories, and recommendations from national and provincial public health expert panels, such as Manitoba's Provincial Vaccine Advisory Committee and the National Advisory Committee on Immunization (NACI). All of these sources of evidence are taken into account to develop recommendations for new vaccine programs and expansions and are weighed by the Province against other competing department initiatives and resources.*	Manitoba Health, Seniors and Active Living, 2019.

*Note.* Serogroup W135 is now called serogroup W.
